# Immunomodulatory interventions in myocardial infarction and heart failure: a systematic review of clinical trials and meta-analysis of IL-1 inhibition

**DOI:** 10.1093/cvr/cvy145

**Published:** 2018-07-14

**Authors:** Mona Panahi, Angelos Papanikolaou, Azam Torabi, Ji-Gang Zhang, Habib Khan, Ali Vazir, Muneer G Hasham, John G F Cleland, Nadia A Rosenthal, Sian E Harding, Susanne Sattler

**Affiliations:** 1 National Heart and Lung Institute, Imperial College London, Du Cane Road, London, UK; 2 Royal Brompton Hospital, Royal Brompton and Harefield NHS Foundation Trust, Sydney Street, London, UK; 3 The Jackson Laboratory, 600 Main Street, Bar Harbor, USA

**Keywords:** Immunomodulation, Immunotherapy, Myocardial infarction, Heart failure, IL-1 inhibition, Systematic review

## Abstract

Following a myocardial infarction (MI), the immune system helps to repair ischaemic damage and restore tissue integrity, but excessive inflammation has been implicated in adverse cardiac remodelling and development towards heart failure (HF). Pre-clinical studies suggest that timely resolution of inflammation may help prevent HF development and progression. Therapeutic attempts to prevent excessive post-MI inflammation in patients have included pharmacological interventions ranging from broad immunosuppression to immunomodulatory approaches targeting specific cell types or factors with the aim to maintain beneficial aspects of the early post-MI immune response. These include the blockade of early initiators of inflammation including reactive oxygen species and complement, inhibition of mast cell degranulation and leucocyte infiltration, blockade of inflammatory cytokines, and inhibition of adaptive B and T-lymphocytes. Herein, we provide a systematic review on post-MI immunomodulation trials and a meta-analysis of studies targeting the inflammatory cytokine Interleukin-1. Despite an enormous effort into a significant number of clinical trials on a variety of targets, a striking heterogeneity in study population, timing and type of treatment, and highly variable endpoints limits the possibility for meaningful meta-analyses. To conclude, we highlight critical considerations for future studies including (i) the therapeutic window of opportunity, (ii) immunological effects of routine post-MI medication, (iii) stratification of the highly diverse post-MI patient population, (iv) the potential benefits of combining immunomodulatory with regenerative therapies, and at last (v) the potential side effects of immunotherapies.

## 1. Introduction

Ischaemic heart disease is estimated to account for 9 million deaths per year and is the leading cause of global mortality.^1^ Importantly, 63% of myocardial infarction (MI) patients in the UK develop heart failure (HF) within 6 years^2^ and ∼90% of mortality post-MI occurs after the development of HF.^3^

The immune system is involved both in events leading up to an MI and in post-MI processes and development towards HF. High levels of systemic inflammation due to metabolic or immunological conditions contribute significantly to an increased risk of cardiovascular disease.^4^^,^^5^ Post-MI, the severe tissue damage caused by an acute infarct is a potent trigger to activate the immune system, which orchestrates the various steps of the post-MI healing process, starting immediately with the removal of cellular debris and restoration of tissue integrity. A tightly controlled balance between inflammatory and regulatory mechanisms is essential, because an inefficient early immune response may lead to fatal cardiac rupture, while excessive inflammation may cause collateral damage to healthy tissue, making both situations detrimental to healing. Once acute inflammation has been cleared, development towards HF may still be exacerbated by a persistent autoimmune-inflammatory condition which maintains low levels of tissue damage sustaining cardiomyocyte loss, fibrosis, and pathological ventricular remodelling.^6^

The clinical relevance of both pre- and post-MI inflammation has been recognized for several decades and a wide variety of clinical trials have attempted to target the immune system, with the majority investigating effects on acute patient survival at a time when the immune response is most evident. With an increased understanding of long-term immunological effects pre- and post-MI, there is growing interest in targeting the immune system to prevent MI and HF. An immense and extensively reviewed body of pre-clinical research^7^^,^^8^ supports the hypothesis that blocking excessive inflammation has beneficial effects on MI prevention and post-MI HF development, yet outcomes of clinical trials to date have been varied and inconclusive as reviewed below.

**Figure 1:**
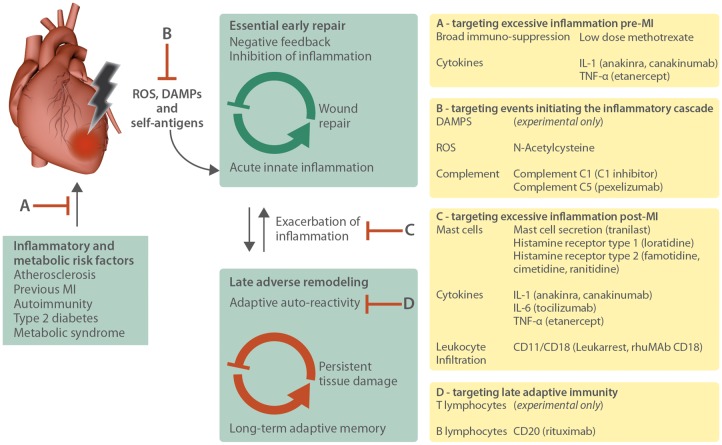
Targets of immunomodulatory clinical trials post-MI. Several underlying metabolic and immunological conditions increase a patient’s risk of suffering a myocardial infarction. The immune response after a myocardial infarct is initiated through the production of ROS and the release of DAMPs from necrotic cardiac tissue. This causes acute innate inflammation crucial for wound repair and the subsequent negative feedback loop to quickly dampen inflammation. However, severe damage causes excessive inflammation and may activate the adaptive immune system, which will persist and cause ongoing tissue damage and may exacerbate adverse remodelling towards heart failure. Clinical trials on immunomodulatory treatment after MI have targeted most aspects of the post-MI immune response. So far the focus has been on preventing MI by targeting inflammatory and metabolic risk factors (*A*), early events initiating the inflammatory cascade (*B*) such as ROS- and DAMP-mediated inflammation and complement-dependent damage, and on attempts to suppress excessive inflammation (*C*) by blocking mast cell function, pro-inflammatory cytokines and leukocyte infiltration. Although still in the minority, some clinical trials have also targeted B and T lymphocytes of the adaptive immune system (*D*).

In this review, we aim to provide a comprehensive overview of clinical trials on pharmacological interventions targeting the immune system to prevent MI or improve post-MI prognosis, from broad immunosuppressive therapy to more refined approaches targeting specific pathways and factors including reactive oxygen species (ROS), complement, mast cells, leucocyte infiltration, inflammatory cytokines interleukin (IL)-1, tumour necrosis factor (TNF), and IL-6, and the inhibition of adaptive B and T lymphocytes (*Figure 1*). Notably, most of these therapeutics are repurposed anti-inflammatory and immunosuppressive agents that have been in long-standing clinical use to prevent transplant rejection or treat autoimmunity.

## 2. Systematic search strategy and meta-analysis methods

A literature search was performed using Preferred Reporting Items for Systematic Reviews and Meta-Analyses (PRISMA) guidelines.^9^ Meta-analysis on selected trials implemented the Grading of Recommendations, Assessment, Development and Evaluations (GRADE) tool^10^ endorsed by the Cochrane collaboration. The review protocol was prospectively registered in PROSPERO.^11^ Briefly, a systematic literature search on immune modulatory trials in MI, HF, and acute coronary syndrome (ACS) patients including a proportion of ischaemia patients, was conducted using PubMed and clinicaltrials.gov. Search terms ‘myocardial infarction’, ‘ventricular remodelling’, ‘ischaemia reperfusion’, and ‘heart failure’ were combined with interventions using simple Boolean operators. Placebo-controlled clinical trials published in the last 50 years, written in English and available as full text online were included in our review.

**Figure 2:**
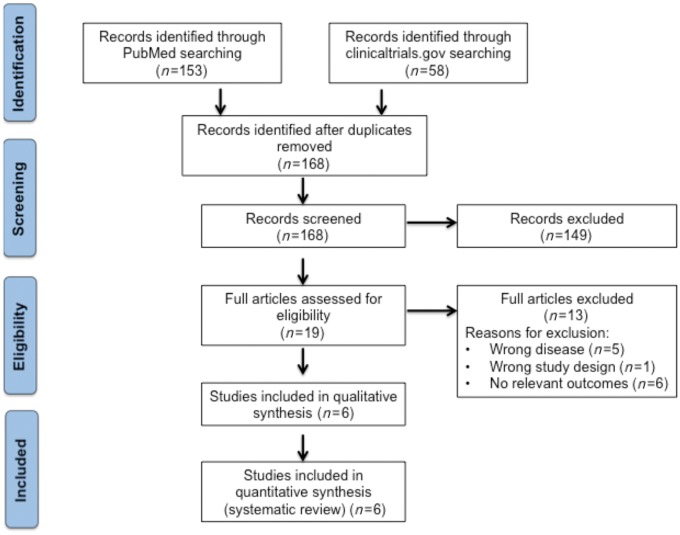
Literature search. Flow diagram of the literature search strategy to identify clinical trials on IL-1 inhibitors suitable for meta-analysis.

Despite a wide range of therapeutic strategies, only clinical trials using IL-1 (anakinra and canakinumab) antagonists have been independently repeated in a way allowing comparison and were selected for meta-analysis to assess treatment efficacy. As shown in *Figure 2*, a total of 168 studies were identified, but only six studies met the above inclusion criteria for qualitative analysis. The Cochrane risk of bias tool was used for the identification of any systematic error or interferences in results to be highlighted. The primary outcome measure analysed was major adverse cardiac events (MACE), which included mortality, incidence of HF, respiratory failure, and acute kidney injury and was reported in three out of six studies. For meta-analyses, results from the three included study were pooled into MedCalc Version 17.5.3^12^ and used to calculate the risk ratios between intervention and placebo, create forest plots, and assess the quality of evidence. The random effects model was implemented using the Dersimonian Laird method.^13^ Random effects models (as opposed to fixed effect models) do not assume one common treatment effect between trials, but each included trial may have a different treatment effect, and they therefore, take into account variance within trials and between trials.^14^

## 3. The post-MI immune response as therapeutic target: from full-blown suppression to targeting specific pathways

Attempts to target the post-MI immune response in clinical trials were first made in the 1960s. Our understanding of the diverse roles of the immune system in tissue homeostasis and the multi-dimensional crosstalk between the immune and the cardiovascular system has increased significantly since that time. An extensive body of pre-clinical research has started to unravel the complex, tightly controlled temporal, and spatial interplay between immune cells and factors in events leading up to MI as well as post-MI,^7^^,^^15^ and it is now appreciated that targeted approaches blocking specific detrimental pathways while maintaining beneficial responses, may be the way forward instead of broad immunosuppression.

### 3.1 Broad-spectrum immunosuppression

Early trials directed at the post-MI immune response used strong broad-spectrum immunosuppressive drugs including corticosteroids, methotrexate, cyclosporin A (CsA), or intravenous immunoglobulin (IVIg). This was based on the hypothesis that all inflammation was detrimental for post-MI recovery.^16^*Table 1* compares study characteristics of clinical trials using broadly immunosuppressive treatment post-MI.

**Table 1:** Study characteristics of clinical trials using broad immunosuppression post-MI

Study	Design	*n*	Study population (% MI)	Intervention	Dose/administration	Follow-up	Outcome measures
**Corticosteroids**							
Donnino *et al.*^17^	R, DB, PC	50	Cardiac arrest (50%)	HC	100 mg IV TDS for 7 days or until shock reversal	7 days	Mortality (0); cumulative vasopressor dose 24 h (0); good neurological outcome (0); shock reversal (0); 24 h IL6 (+); 24 h IL10 (0)
Liu and Liu^18^ (COPE-ADHF)	R, DB, PC	102	CHF (26.5%)	D, P	20 mg IV od; 1 mg/kg daily (max 60 mg) for 7 days then tapered off	19 months	Mortality (+); 30/7 cardiac mortality (+); urine output (+); serum creatinine 7/7 (+)
Mentzelopoulos *et al.*^19^	R, DB, PC	268	Cardiac arrest (20%)	MP, HC	40 mg IV once, 300 mg IV daily for 7/7 (gradually tapered)	2 months	Mortality (+); good neurological outcome (+); ROSC (+)
Tsai *et al.*^20^	NR, OL, PC	97	Cardiac arrest (32%)	HC	100 mg IV during resuscitation	7 days	Mortality(0); Discharge date (0); ROSC (+)
Giugliano *et al.*^16^							
Madias (1982)	R, DB, PC	40	Acute MI	MP	2 g once, then repeat 3 days later	6 months	Mortality (0); ECG Changes (0); MACE (0)
Henning (1981)	R, DB, PC	28	Acute MI	MP	30 mg/kg IV twice (after initial measurements and 2 days after)	2 days	Mortality (0); cardiac index (+); cardiac output (+); infarct size (0); mean arterial pressure (0); systemic vascular resistance (+); urine output (+); CK (0); lactate (−)
Peters (1978)	R, DB, PC	29	Acute MI	MP	15 mg/kg IV 7 h and 17 h post-MI and 30 mg/kg two doses IV 7 h and 10 h	70 h	Congestive heart failure development (0); infarct size (0); CK (0); CK-MB (0)
Barzilai (1972)	NR, OL, NPC	937	Acute MI	HC	500 mg IV daily for 3 days, then 500 mg infusion over 8–10 days	3 weeks	Mortality (+)
**Methotrexate**							
Moreira *et al.*^21^ (TETHYS)	R, DB, PC	84	STEMI	Methotrexate	0.05 mg/kg+ 0.05 mg/kg/h for 6 days	3 months	Mortality (0); coronary blood flow (0); infarct size (0); LVEF (−); MACE (0); TIMI (0); BNP (0); CK (0); CK-MB (0); troponin (0); Hs-CRP (0); ESR (0)
Everett *et al.*^22^ (CIRT)	R, DB, PC	7000	Inflammatory cardiovascular disease	Methotrexate	15–20 mg/week	3–5 years	Mortality (TBC); MACE (TBC)
Moreira *et al.*^23^ (METIS)	R, DB, PC	50	CHF	Methotrexate	7.5 mg/week for 12 weeks	3 months	MACE (0); 6MWT (0); NYHA (0); CRP (0)
Gong *et al.*^24^	R, SB, PC	62	CHF	Methotrexate	7.5 mg/week for 12 weeks	3 months	LVEDD (0); LVEF (0); 6MWT (+); NYHA (+); QOL score (+); CRP (+); sICAM-1 (+); sIL-1RA (+); IL-10 (+); IL-16 (+); MCP-1 (+); TNF-α (+)
**Cyclosporine A**							
Hausenloy *et al.*^25^	R, DB, PC	78	Elective CABG	CsA	2.5 mg/kg IV	3 days	MACE (0), peri-operative myocardial injury in CABG (+); CK-MB (0); cTnT (0).
Chiari *et al.*^26^	R, SB, PC	61	Peri-operative MI	CsA	2.5 mg/kg IV	3 days	MACE (0); cTnI (+).
Yingzhong *et al.*^27^							
Ottani (2016) (CYCLE)	R, OL, PC	410	AMI	CsA	2.5 mg/kg IV	6 months	Mortality (0); ECG ST resolution (0); LV A/D (0); LVEF (0); MACE (0); hs-TNT (0).
Cung (2015)	R, DB, PC	681	AMI (STEMI)	CsA	2.5 mg/kg IV	1 year	Mortality (0); ECG (0); LVEDV (0); LVEF (0); LVESV (0); MACE (0); CK (0).
Ghaffari (2013)	R, DB, PC	101	STEMI	CsA	2.5 mg/kg IV	6 months	Mortality (0); Infarct size (0); LVEF (0); MACE (0).
Mewton (2010); Piot (2008)	R, SB, PC	58	AMI	CsA	2.5 mg/kg IV	6 months	EDWT (0); Infarct size (+); LVEDV (0); LVEF (0); LVESV (+); MACE (0); CK (+); TnI (+).
**IVIg**							
Gullestad *et al.*^28^	R, DB, PC	62	Acute MI	IVIg	0.4 mg/kg od for 5 days then 0.4 mg/kg monthly for 5 months	6 months	LVEDV (0); LVEF (0); LVESV (0); scar area (0); ILRA (+); IL-10 (0); lymphocytes (+); MCP-1 (−); neutrophils (+); TNFα (−), TNFR1 (+).
Gullestad *et al.*^29^	R, DB, PC	40	CHF (57.5%)	IVIg	0.4 g/kg od for 5/7 then 0.4 g/kg monthly for 5 months	6 months	IL1β (+); IL-1RA (+); IL-10 (+); TNF-αR (+); LVEF (+); N-ANP (+); TNF-α (0)

6MWT, 6 min walk test; BNP, B-natriuretic peptide; CHF, chronic heart failure; CsA, cyclosporine A; CK, creatine kinase; CK-MB, creatine kinase-MB; D, dexamethasone; DB, double blind; ECG, electrocardiogram; ESR, erythrocyte sedimentation rate; HC, hydrocortisone; hs-CRP, high-sensitivity C-reactive protein; IL, interleukin; IL-RA, interleukin receptor antagonist; IV, intravenous; IVIg, intravenous immunoglobulin; LVEDD, left ventricular end-diastolic diameter; LVEDV, left ventricular end-diastolic volume; LVEF, left ventricular ejection fraction; LVESV, left ventricular end-systolic volume; MACE, major adverse cardiac event; MCP-1, monocyte chemotactic protein-1; MP, methylprednisolone; N-ANP, n-terminal pro-atrial natriuretic peptide; NPC, not placebo controlled; NR, non-randomized; NYHA, New York Heart Association; OL, open label; P, prednisolone; PC, placebo controlled; PCI, percutaneous coronary intervention; QOL, quality of life score; R, randomized; ROSC, return of spontaneous circulation; SB, single blind; sIL-1RA, secreted interleukin-receptor antagonist; STEMI, ST-elevation myocardial infarction; TBC, to be confirmed in future study; TDS, three times a day; TNF-a, tumour necrosis factor alpha; TNFR1, tumour necrosis factor receptor Type 1; (þ), supports intervention use; (−), supports placebo use; (0), no difference between intervention and placebo.

#### 3.1.1 Corticosteroids

Corticosteroids are in well-established clinical use to treat inflammatory conditions such as allergies and autoimmunity. Their use post-MI was expected to be beneficial, due to their overall suppressive effect on the recruitment and activation of both innate and adaptive immune cells.^30^ However, despite pre-clinical and clinical research for over 50 years, evidence concerning the efficacy of corticosteroids after MI is still conflicting.

A previous systematic review with meta-analysis assessed corticosteroid use in 2646 patients with acute MI (AMI) in 11 trials from 1964 to 1989.^16^ Although corticosteroids decreased mortality by 26% compared to placebo, this did not seem to stem from a cardio-protective effect, as infarct size or myocardial rupture rates were unchanged. Meta-analysis findings were limited, as most included studies were small (≥40 patients) and little information regarding protocol and measurements had been provided. Furthermore, very short follow-up periods (median 24.5 days) limited the possibility to assess any effects on development of post-MI HF.

More recent results of subsequent corticosteroid trials are still conflicting, but due to high diversity in study populations, the varied nature of clinical endpoints and different follow-up periods, meaningful comparisons are hard to draw. However, overall mortality was reported to be decreased in only two out of four studies performed since publication of the previous meta-analysis. Importantly, decreased mortality was reported in trials with slightly longer follow-up periods, while acute studies fail to show improvement. The COPE-ADHF trial (2013) reported significant improvement in mortality with dexamethasone followed by prednisolone treatment for 7 days in acute decompensated HF (27% AMI). This benefit persisted over the follow up period of 1–36 months, and the number of deaths was three in the steroid as compared with 10 in the placebo group.^18^ A hydrocortisone trial by Tsai *et al.*^20^ in 97 non-randomized cardiac arrest patients also improved haemodynamics by 40%, without increased survival rates. A similar trial by Mentzelopoulos *et al.*^19^ in which cardiac arrest patients were administered methylprednisolone during cardiopulmonary resuscitation followed by hydrocortisone, showed increased survival until hospital discharge, and improved haemodynamics and organ function during a 1 year follow-up. However, hydrocortisone did not affect cardiac shock reversal or mortality rates in another randomized control trial by Donnino *et al.*^17^ with 50 cardiac arrest patients. 

#### 3.1.2 Methotrexate

Methotrexate is another immunosuppressive drug commonly used in clinical practice for the treatment of rheumatoid arthritis. Its mechanism of action involves the release of endogenous adenosine, and suppression of innate inflammation and the adaptive immune response.^31^ The addition of methotrexate to conventional chronic HF (CHF) therapy was supported by a small trial by Gong *et al.*^24^ in 2006 including 71 patients who received either a 7.5 mg weekly dose of methotrexate or placebo over a 12 week period. This improved patients’ inflammatory profile including levels of IL-6, TNF, monocyte chemoattractant protein-1, and C-reactive protein (CRP), New York Heart Association (NYHA) functional group, quality of life and 6 min walk test (6MWT) distance when compared with placebo. Conversely, the METIS trial (2009) using a similar treatment regime in CHF patients found no improvement in 6MWT or CRP, and although a trend was shown towards improved the NYHA group, differences were not significant.^23^ The TETHYS trial of methotrexate (2017) in 84 ST-Elevation MI (STEMI) patients on the other hand showed worsened left ventricular ejection fraction (LVEF) and no reduction in infarct size at 3 months follow-up compared with the placebo group.^21^

Low-dose methotrexate is currently also being tested for its usefulness in the prevention of cardiovascular morbidity and mortality in 7000 patients with prior MI and Type 2 diabetes or metabolic syndrome (Cardiovascular Inflammation Reduction Trial, CIRT).^22^ CIRT aims to test the inflammatory hypothesis of atherothrombosis, which suggests that inhibition of inflammation may reduce atherothrombosis and thus prevent occurrence of adverse cardiovascular events.

#### 3.1.3 Cyclosporin A

CsA inhibits antigen presentation by dendritic cells and effector T lymphocyte function and has been in long-standing clinical use as immunosuppressant to block transplant rejection.^32^ Notably, however, use of CsA post-MI was attempted due to its ability to protect cardiomyocytes from cell death due to ischaemia/reperfusion injury (I/RI). This cardio-protective function of CsA stems from its ability to inhibit the opening of the mitochondrial permeability transition pore (mPTP) and thereby prevent necrotic cell death.^33^ A meta-analysis by Yingzhong *et al.*^27^ assessed the use of CsA in five randomized control trials of AMI patients, but the authors did not find any difference in all-cause mortality, LVEF, or infarct size possibly due to small sample sizes and short follow-up periods. Hausenloy *et al.*^25^ found no difference in cardiac troponin T (cTnT) levels between CsA and the placebo group in 78 elective coronary artery bypass graft (CABG) patients, over half of which were post-MI patients. However, follow-up was only 3 days as the focus was on peri-operative myocardial injury rather than long-term HF development. Notably, a significant reduction in peri-operative myocardial injury was observed in higher-risk patients with longer cardiopulmonary bypass times, with CsA therapy, with a reduced post-operative cTnT rise when compared with control. In a similar study, Chiari *et al.*^26^ found reduced cardiac troponin I (cTnI) but this change was not reflected in patients’ clinical function 3 days after infarct.

As these trials investigated acute effects of CsA on immediate post-MI cardiomyocyte death, follow-up periods were very short, and immunosuppressive effects on dendritic cell activation and effector T lymphocyte function were not taken into account. Therefore, analysis of long-term effects of CsA treatment on the adaptive immune response and development of HF would be informative.

#### 3.1.4 IVIg

IVIg is intravenously administered immunoglobulin G obtained from pooled donor plasma. It binds to and neutralizes components of the immune system which could otherwise instigate downstream inflammatory effects. This includes necrotic debris, danger-associated molecular patterns (DAMPs), complement, integrins, cytokines, and leucocyte receptors.^34^ IVIg is used clinically in a range of immunological and inflammatory diseases, either as replacement therapy for antibody deficiencies or as immunosuppressive for inflammatory/autoimmune conditions.^35^

A potentially beneficial effect of IVIg in the context of cardiovascular disease was first tested by Gullestad *et al.*^29^ in 40 patients with stable CHF, 23 of which suffered from post-ischaemic damage, and LVEF was <40%. Patients received either IVIg or placebo for 21 weeks. At 6 months follow-up, IVIg significantly up-regulated the anti-inflammatory cytokine profile and increased LVEF by 5% compared with baseline. However, when the same team repeated this treatment in 62 patients with AMI following percutaneous coronary intervention (PCI), there was no difference between the IVIg and the placebo groups.^28^

In summary, although broad immunosuppression by corticosteroids, methotrexate, CsA, and IVIg are widely accepted in clinical practice as gold-standard anti-inflammatory and immunosuppressive therapies, their use immediately following MI remains controversial. Beneficial effects in clinical trials are inconsistent and some studies suggest detrimental effects on the clearance of necrotic cardiomyocytes and disrupt post-infarct healing after AMI, causing concerns about a large risk-benefit ratio.^36^^,^^37^ Importantly, currently available information indicates a potentially beneficial effect of immunosuppression during HF or possibly used as preventive strategy, while treatment of AMI patients showed no effects or worsened outcomes. This discrepancy is likely to reflect the opposing role of the immune system immediately after AMI and during CHF. While the acute immune response after AMI initiates reparative processes and is essential to restore tissue integrity, persistent inflammation and autoimmune-mediated tissue damage may be detrimental in CHF. In addition, if events leading up to AMI are mediated or exacerbated by inflammation, low-dose immunosuppressive treatment may also be beneficial in the prevention of AMI. However, if considered as long-term treatment to prevent AMI or development towards HF, dosing of immunosuppressive regimes needs to be assessed carefully to avoid serious adverse effects including infections and cancers.

### 3.2 Targeting selected pathways to achieve immunomodulation

Recently, our understanding of the complexity of immune pathways involved in early repair as well as long-term pathogenesis after MI has improved significantly. The necessity of early acute inflammation and the need for a balanced and co-ordinated response for successful healing and functional recovery are now being appreciated. A wide range of studies have therefore aimed to target individual pathways to modulate the immune response towards regeneration instead of fully suppressing the immune system.

#### 3.2.1 Immediate post-ischaemic events

Acute I/RI injury immediately after infarct is mediated by the production of ROS and mitochondrial dysfunction which causes necrotic cell death and the release of DAMPs. DAMPs are intracellular molecules passively released during myocardial necrosis and fragments of the extracellular matrix. They include prominent examples such as hyaluronic fragments, high-mobility group box 1, heat-shock proteins, and act as potent inflammatory activators for most cell types including cardiomyoctes, fibroblasts, endothelial cells, and immune cells.^15^ DAMPs can trigger expression of pro-inflammatory cytokines and pro-hypertrophic proteins, initiate cell death pathways, promote endothelial dysfunction, and induce fibrosis.^38^ So far, clinical trials aiming at events immediately after MI have targeted early inflammatory mediators such as mitochondrial damage, as discussed above, ROS, and the complement system (*Table 2*).

**Table 2:** Study characteristics of clinical trials targeting early inflammation post-MI

Study	Design	*n*	Study population	Intervention	Dose/administration	Follow-up	Outcome measures
**N-Acetylcysteine**							
Pasupathy *et al.*^39^ (NACIAM)	R, DB, PC	112	STEMI	NAC (+NTG)	20 mg/min/1 h then 10 mg/min for 47 h (total 29 g over 2 days)	3 months	EDV (0); ESV (0); Infarct size (+); LVEF (0); MVO (0); Myocardial salvage (+); SV (0); symptomatic (+)
Yesilbursa *et al.*^40^	R, DB, PC	22	AMI	NAC (+NTG+ streptokinase)	15 g IV infused over 24 h	3 months	DF (0); DT (0); *E*/*A* ratio (0); LVEDD (+); LVEF (+); LVESD (+); LVWMSI (+); SF (0); CK-MB (0); plasma MDA (+)
ŠOchman *et al.*^41^ (ISLAND)	R, BU, PC	30	AMI	NAC (+NTG + streptokinase 1.5 milU +aspirin 200 mg +2.5–5 mg midazolam)	100 mg/kg IV (50 mg/kg undiluted bolus; 50 mg/kg in 200 ml IV saline over 30 min)	2 weeks	CK (+); ECG 0; LVEF (+); MACE (+); REF (+)
Arstall *et al.*^42^	R, BU, NPC	25	AMI	NAC (+NTG, + streptokinase)	15 g IV infused over 24 h	7 days	ECG (0); LVEF (0); GSH (+), GSH/GSSG ratio (+), MDA (+).
**Complement inhibitors**							
Fattouch *et al.*^43^	R, DB, PC	80	STEMI	C1-INH	1000 UI	48 h	Mortality (0); CI; CVP (0); GRMK (+); MAP (+); MPAP (0); SV (+); PCWP (0); cTnI (+); PCR (0); SVR (0)
Thielmann *et al.*^44^	R, OL, PC	67	STEMI	C1-INH	40 IU/kg bolus + 20 IU/kg	30 days	C3c/C4 levels (+); cTnI (+)
Zwaan *et al.*^45^	NR, OL, NPC	22	STEMI	C1-INH	50 U/kg + 1.25 U/kg/h for 48 h OR 100 U/kg + 1.25 U/kg/h for 48 h	3 days	C4 (+); CK-MB (+); cTnT (+)
Testa *et al.*^46^							
APEX AMI Investigators (2007)	R, DB, PC	5745	STEMI	Pexelizumab	2 mg/kg + 0.05 mg/kg per/h for 24/24	3 months	Mortality (0); MACE (0)
Smith (2006) (PRIMO-CABG II)	R, DB, PC	4254	Elective CABG	Pexelizumab	2 mg/kg + 0.05 mg/kg/h for 24/24	6 months	Mortality (0); MACE (0);
Smith (2004) (PRIMO-CABG I)	R, DB, PC	3099	Elective CABG	Pexelizumab	2 mg/kg + 0.05 mg/kg/h for 24/24	6 months	Mortality (+); MACE (+); CK-MB (+)
Mahaffey (2003) (COMPLY)	R, DB, PC	943	STEMI	Pexelizumab	2 mg/kg OR 2 mg/kg + 0.05 mg/kg/h for 20/24	6 months	Mortality (0); ECG (0); MACE (0); CK-MB (0)
Granger (2003) (COMMA)	R, DB, PC	960	STEMI	Pexelizumab	2 mg/kg OR 2 mg/kg + 0.05 mg/kg/h for 20/24	6 months	Mortality (+); ECG (0); MACE (0); CK-MB (0)
Shernan (2004)	R, DB, PC	914	Elective CABG	Pexelizumab	2 mg/kg OR 2 mg/kg + 0.05 mg/kg/h for 20/24	7 days	Mortaily (0); composite score (0); ECG (0); MACE (0); future MI (+); LV dysfunction (0); CK-MB (0)

C1-INH, complement 1 protein inhibitor; C3/C4, complement protein 3 and 4; CABG, coronary artery bypass graft; CI, cardiac index; CK, creatine kinase; CK-MB, creatine kinase-MB; cTnI, cardiac troponin I; cTnI/T, cardiac troponin I/T; DB, double blind; DF, diastolic flow; DT, deceleration time; E/A ratio, early atrial filling ratio; ECG, electrocardiogram; EDV, end-diastolic volume; ESV, end-systolic volume; GSH, glutathione; GSH/GSSG, glutathione/ oxidized glutathione ratio (redox status); GRMK, global and regional myocardial kinesia; IV, intravenous; IVRT, isovolumic relaxation time; LVEDD, left ventricular end-diastolic diameter; LVEF, left ventricular ejection fraction; LVWMSI, left ventricular wall motion index score; MACE, major adverse cardiac event; MAP, mean arterial pressure; MDA, malondialdehyde; MI, myocardial infarction; MVO, microvascular obstruction; OL, open label; PC, placebo controlled; NAC, N-acetylcysteine; NPC, not placebo controlled; NR, non-randomized; NTG, nitroglycerin; PCWP, pulmonary capillary wedge pressure; R, randomized; REF, regional ejection fraction; SF, systolic flow; STEMI, ST-elevation myocardial infarction; SV, stroke volume; UI, international units; (þ), supports intervention use; (−), supports placebo use; (0), no difference between intervention and placebo.

##### ROS

ROS are highly reactive chemical compounds including peroxides, superoxide, and nitric oxide, which promote leucocyte recruitment into the damaged tissue and cause further oxidative stress, resulting in more macromolecular damage.^47^ This supports the use of anti-oxidants such as N-acetylcysteine (NAC) to block ROS post-MI^48^ and evidence of beneficial effects of NAC was first demonstrated in a small trial in 27 MI patients by Arstall *et al.*^42^ Although no functional changes were established by echocardiography, significantly less oxidative stress was observed in NAC treated patients. In two other early trials with small study populations, ISLAND (1996) and Yesilbursa *et al.*,^40^^,^^41^ NAC treatment improved LV function and reduced oxidative stress post-MI. Benefits of NAC use were confirmed by the NACIAM (2017) trial of 112 STEMI patients undergoing PCI, which received NAC and nitrate therapy. On cardiac magnetic resonance imaging (cMRI), high-dose NAC doubled myocardial salvage from 27% to 60% and an absolute reduction in infarct size by 5.5% compared with placebo was observed.^39^ However, it is impossible to speculate about the long-term effects of this intervention, as patients were observed only 7 days after treatment.

Decades of research and several small clinical trials now provide a promising base for a large randomized multi-centric study, to finally answer the question if NAC treatment is a beneficial agent to reduce reperfusion injury after MI. If designed with long-term effects in mind, benefits related to HF development may be assessed as well. Importantly, when interpreting NAC trial results, it also needs to be considered that anti-oxidants affect other molecular pathways too and beneficial effects may not be due to immunological effects only.^49^

##### Complement

The complement system is part of innate immunity and a complex network of proteins in the blood responsible for clearance of microbes and damaged cells. Upon reperfusion of occluded vessels, tissue damage triggers complement activation leading to inflammation, and additional tissue destruction. In particular, the classical complement pathway is activated and can be chronically sustained, which directly damages the myocardium.^50^

*C1:* The C1 proteins initiate the classical complement cascade. Inhibition of the C1 receptor using C1-INH in AMI patients given thrombolytic therapy showed decreased cTnT and Creatine Kinase-MB (CK-MB) levels.^45^ In a study of 67 STEMI patients undergoing emergency CABG, Thielmann *et al.*^44^ showed that C1-INH significantly reduced C3c/C4 and cTnI levels. Similarly, in 80 STEMI patients undergoing emergency CABG, Fattouch *et al.*^43^ described improvement in functional measures and overall survival. Cardiopulmonary bypass support, high-dose inotropes and time of intubation, intensive care unit stay, and in-hospital stay were all greatly reduced in the C1-INH group. Furthermore, these patients showed significantly improved mean arterial pressure, cardiac index, stroke volume, and cTnI levels.

*C5:* C5 is part of the classical complement cascade acting downstream of C1. A previous meta-analysis on clinical trials using pexelizumab, a monoclonal antibody against C5, showed no improvement in outcomes following MI, but reduced mortality in patient undergoing CABG.^46^

*Table 2* summarizes study characteristics of trials targeting early inflammation post-MI by blocking ROS and complement. Although more thorough studies are required to reach conclusive results, the above described clinical trials provide promising results that early post-MI events such as ROS- and complement-mediated damage may be potential targets to improve post-MI outcome. Notably, therapies targeting events immediately after AMI are restricted by a brief window of opportunity after initial myocardial damage or restoration of blood flow, and treatment needs to be timed accurately.

#### 3.2.2 Leucocyte infiltration

Another approach to prevent excessive inflammation and associated tissue destruction post-MI is to prevent immune cells from infiltrating the damaged tissue (*Table 3*). Acute damage causes local inflammation, activation of endothelial cells in the vasculature and up-regulation of adhesion molecules such as integrins to recruit leucocytes. Neutrophils are among the first leucocytes to migrate into the myocardium. They clear cellular debris, promote macrophage infiltration, and are essential for cardiac repair and survival.^60^ Canine studies showed that neutrophil depletion reduces infarct size,^61^ however, post-MI neutropenia could delay cardiac repair and preventing their infiltration is associated with decreased survival in mice.^62^

**Table 3:** Study characteristics of clinical trials targeting innate immune cells

Study	Design	*n*	Study population	Intervention	Dose/administration	Follow-up	Outcome measures
**CD11/CD18 integrin inhibitors**							
Faxon *et al.*^51^ (HALT-MI)	R, DB, PC	420	STEMI	Hu23F2G (Leukoarrest)	0.3 mg/kg OR 1 mg/kg IV bolus	1 month	Mortality (0); Infarct size (0); MACE (0).
Rusnak *et al.*^52^ (FESTIVAL)	R, DB, PC	60	Anterior or inferior STEMI (AMI)	Hu23F2G (Leukoarrest)	0.3 mg/kg OR 1 mg/kg IV bolus	6 weeks	Infarct size (0); MACE (0); physical exam (0); Vital signs (0)
Baran *et al.*^53^ (LIMIT AMI)	R, DB, PC	394	STEMI	rhuMAb CD18	0.5 mg/kg OR 2 mg/kg IV bolus	3 months	Mortality (0); coronary blood flow (0); ECG STEMI resolution (0); infarct size (0); MACE (0); TIMI (0); CK-MB (0).
**Mast cells**							
Holmes *et al.*^54^ (PRESTO)	R, DB, PC	11 484	PCI (40%)	Tranilast	300 mg OR 450 mg twice daily oral for 1 month OR 3 months	9 months	Mortality (0); MACE (0); MI (0), target vessel revascularization (0).
Tamai *et al.*^55^ (TREAT 2)	R, DB, PC	288	Angina with previous MI (52.1%) with successful PTCA and de novo or restenotic coronary lesions	Tranilast	600 mg daily oral for 3/12	12 months	MACE (0); restenosis (+); SAE (−). Target vessel revascularization (0).
Tamai *et al.*^56^ (TREAT)	R, DB, PC	247	Angina with previous MI (39.7%) with successful PTCA and de novo lesions	Tranilast	600 mg OR 300 mg daily oral for 3/12	12 months	MACE (0); restenosis (+); side effects (0); target vessel revascularization (0);
Mujtaba *et al.*^57^	R, BU, PC	60	STEMI	H2RA (famotidine)	40 mg daily	1 month	LVEF (+); LV dilation (+); Nt-pro-BNP (+).
Kim *et al.*^58^	R, DB, C and R, OL, prospective	50	CHF	H2RA (famotidine)	30 mg daily for 6 months	6 months	BNP (0); fractional shortening (0); LV diameter (0); LVDd (0); LVDs (0); LVEDV (+); LVESV (+); MACE (+).
Lucas *et al.*^59^	R, DB, PC4-way crossover	12	CHF (NYHA II and III) (50%)	H2RA (cimetidine, famotidine, ranitidine)	400 mg twice daily cimetidine and 40 mg famotidine daily and 150 mg daily ranitidine for 7 days each with a 7 day washout between each intervention	7 days	aerobic metabolic performance (0); exercise capacity (0); LVEF (0).

BNP, B-natriuretic peptide; BU, blinding unknown; CHF, chronic heart failure; D, dexamethasone; DB, double blind; H2RA, histamine-2 receptor antagonist; IV, intravenous; LVDd, left ventricle dimensions in diastole; LVDs, left ventricle dimensions in systole; LVEDV, left ventricular end-diastolic volume; LVEF, left ventricular ejection fraction; LVESV, left ventricular end-systolic volume; MAb, monoclonal antibody; MACE, major adverse cardiac event; NYHA, New York Heart Association; OL, open label; PC, placebo controlled; PCI, percutaneous coronary intervention; PTCA, percutaneous transluminal coronary angioplasty; R, randomized; SAE, serious adverse events; STEMI, ST-elevation myocardial infarction; TIMI, thrombolysis in myocardial infarction; (þ), supports intervention use; (−), supports placebo use; (0), no difference between intervention and placebo.

##### Integrins

Infiltration of leucocytes into damaged or inflamed tissues is mediated by adhesion molecules on activated endothelial cells. Up-regulation of cardiac α_V_β_3_ integrin at recently infarcted sites (measured 2 weeks after AMI) predicts improved repair and recovery in human patients,^63^ and blocking P-selectin and intercellular adhesion molecule-1 (ICAM-1) in murine studies is beneficial post-MI.^64^ This supported the notion that inhibition of leucocyte infiltration might improve post-MI outcomes.

Although treatment with monoclonal antibodies against the integrin complex CD11/CD18 has reduced infarct sizes in pre-clinical studies, the corresponding clinical investigations in AMI patients have not been encouraging. The LIMIT-AMI study (2001) targeted CD18 in a study of 394 patients, but did not find any difference in coronary blood flow, infarct size, or the rate of ECG ST-segment elevation resolution.^53^ Similarly, the FESTIVAL study (2001) tested a monoclonal antibody against CD11/CD18 in 60 patients following coronary angioplasty and did not find any difference in infarct size at 30 day follow-up.^52^ There was also no improvement in infarct size measured 5–9 days following an anti-CD11/CD18 receptor antibody at either high or low dose in a 420 participant study in the HALT-MI (2002) investigation.^51^ These studies all measured infarct size or flow rate as primary outcomes. A trial measuring effects of anti-CD11/CD18 treatment on heart function would be informative.

##### Mast cells

The number of cardiac mast cells increases dramatically following MI, along with macrophages, and they influence the inflammatory process via histamine, growth factor, and cytokine release.^65^ They also induce fibroblast differentiation and modulate myocardial tissue healing and formation of the infarct scar.^66^

Tranilast is an anti-fibrotic drug which prevents collagen synthesis by inhibiting the release of mast cell factors, including histamine, and prostaglandins.^67^ Two early trials (TREAT and TREAT 2) showed that 600 mg tranilast daily for 3 months caused a significant reduction in restenosis required for non-stented patients. On angiography, the TREAT trial (1999) reported a restenosis rate of 14.7% compared with 46.5% in the placebo group, and 18.8% compared with 44.1% in the TREAT-2 trial (2002).^55^^,^^56^ However, the PRESTO trial (2002) failed to show any improvement in quantitative measures of restenosis as measured by angiography and intravascular ultrasound in 11 484 patients treated with tranilast twice daily for 1 or 3 months, 4 h following PCI. There was no difference to placebo treatment in mortality, MI, or ischaemia-driven target vessel re-vascularization in the 9 month follow-up.^54^

Histamine released by cardiac mast cells acts on two distinct histamine receptors type 1 and 2 (H1R and H2R), which increase endothelial permeability and exacerbate ischaemia/reperfusion injury I/RI, resulting in increased infarct size.^68^^,^^69^ A small single-blind trial by Mujtaba *et al.*^57^ with 60 STEMI patients prescribed 40 mg daily H2R antagonist famotidine upon hospital admission showed reduced LV dilation. However, LVEF was also decreased on echocardiography when followed up at day 30. Another trial by Kim *et al.*^58^ on 318 CHF patients, who received 30 mg famotidine daily for 6 months, showed reduced ventricular remodelling with a reduction in LV end-diastolic and end-systolic diameters, lower brain natriuretic peptide (BNP), and decreased NYHA functional class. Conversely however, a small study on 12 patients by Lucas *et al.*^59^ using three H2R antagonists (H2RA) cimetidine, famotidine and ranitidine administered for 7 days did not improve metabolic or exercise performance or LVEF upon echocardiography compared with placebo in NYHA Class II/III patients.

Thus far, clinical trials using early leucocyte accumulation as therapeutic target have included non-specific blocking of leucocyte migration by inhibiting the integrin complex CD11/CD18 and blocking of mast cell effector functions (*Table 3*). While the first approach has not yielded any beneficial effects, targeting mast cells seems a promising avenue worth exploring further. Importantly, as for ROS and complement targeting strategies, treatments need to be administered at the best time to suppress excessive infiltration, while maintaining healing responses and beneficial cardiac remodelling. This likely means a delay of immunomodulatory treatment of 2–3 days to maintain acute neutrophil and monocyte infiltration to support the initiation of the healing process, while blocking persistent infiltration and excessive accumulation within the tissue once the acute response should be cleared.

#### 3.2.3 Adaptive immunity

MI not only triggers acute early inflammation, but also a substantial adaptive immune response against cardiac self-proteins. Due to the immunological memory of adaptive immune cells, this auto-reactivity in form of self-reactive T and B lymphocytes and auto-antibodies lingers, and has been suggested to cause ongoing tissue destruction, exacerbating the risk of developing HF.

##### T lymphocytes

Pre-clinical studies have shown that T lymphocytes accumulate in the infarct zone within minutes following I/RI and that CD4^+^ T lymphocytes contribute to additional tissue injury.^70^

As discussed above, CsA was used as experimental post-MI treatment due to its role in blocking mPTP opening, and was therefore, expected to reduce mitochondrial dysfunction and cardiomyocyte death immediately after AMI. However, CsA also targets calcineurin to inhibit the transcription of IL-2, IL-4, and CD40, cytokines and co-receptors important for effector T lymphocyte recruitment and function.^71^ Trials performed so far did not find any difference in cardiac damage as measured by cTnT levels, LVEF, infarct size, or all-cause mortality three days after infarct.^25–27^ These studies focused on the effects of CsA administration immediately after infarct. However, the adaptive immune system remains active long-term after MI. Therefore, considering that all-cause mortality did not change and CsA treatment was deemed safe, longer treatments and in particular longer follow-up periods may be worthwhile to investigate any long-term effect on cardiac remodelling and function.

The pro-inflammatory activity of effector T lymphocytes and other inflammatory immune cells is counterbalanced by the regulatory arm of the immune system, most prominently by T regulatory (Treg) cells. Experimental studies indicate an important role of Treg cells in improving post-MI tissue regeneration by modulating the function of other leucocytes.^72^ Treg cells have anti-inflammatory properties which promote skeletal muscle regeneration, and might therefore, also establish a pro-regenerative environment in the heart. Boosting the Treg cell population has been proven beneficial in a variety of inflammatory settings and might be a therapeutic avenue worth exploring in post-MI patients.^73^ Adenosine, a naturally occurring nucleoside increases Treg cell numbers,^74^ and although the rationale for its use post-MI was to protect cardiomyocytes from acute I/RI, it has also been shown to reduce infarct size in pre-clinical studies by suppressing CD4^+^effector T lymphocyte subsets.^70^ A meta-analysis of 15 randomized-controlled trials involving 1736 patients with AMI undergoing PCI reported a reduction in HF development with adenosine use, but there was no overall improvement in LVEF or survival.^75^ Confirmation of any effects on the adaptive immune system would be informative. No clinical trials specifically targeting the Treg cell population have been performed yet.

##### B lymphocytes

DAMPs and damaged myocardial tissue activate circulating CD20^+^B lymphocytes. These selectively produce the chemokine CCL7, which causes inflammatory monocytes to migrate from the bone marrow into the circulation. High circulating levels of CCL7 and B cell activating factor (BAFF) were associated with higher rate of mortality in patients with recurrent MI. Antibody-mediated depletion of CD20 or BAFF in experimental MI models reduced inflammatory recruitment and improved cardiac function.^76^

Rituximab is an anti-CD20 antibody used clinically to treat inflammatory autoimmune diseases and B lymphocyte cancers. The RITA-MI study is currently recruiting participants to test its clinical efficacy and safety in AMI.^77^

#### 3.2.4 Inflammatory cytokines

A long list of cytokines and chemokines has been implicated in post-MI inflammation, but the best studied cytokine targets post-MI to date are IL-1, IL-6, and TNF. Study characteristics for clinical trials on cytokine inhibition are shown in *Table 4*.

**Table 4:** Study characteristics of clinical trials using cytokine inhibition

Study	Design	*n*	Study population (% ischaemia)	Intervention	Dose/administration	Follow-up	Outcome measures
**IL-1 inhibitors**							
Ridker *et al.*^78^ (CANTOS)	R, DB, PC	10 061	Previous MI	Canakinumab	150 OR 200 OR 300 mg	3.7 years	Mortality (0); MACE (+); SAE (−); CRP (+); LDL (0).
Van Tassell *et al.*^79^	R, DB, PC	30	HF (33.3%)	Anakinra	100 mg twice daily for 3 days + daily for 11 days	2 weeks	MACE (0); clinical cardiac exam (0); LVEF (+); CRP (+); IL-6 (+); IL17 (0); lpPLA2 (0); Leptin (0); MPO (+); NTpro-BNP (0); galectin 3 (0); TNFa (0); hs-TnI (+).
Morton *et al.*^80^ (MRC-ILA Heart Study)	R, DB, PC	182	ACS (26%)	Anakinra	100 mg sc once only	1 year	Mortality (0); MACE (−); hs-CRP (+); FBC (0); IL-6 (+); platelets (+); TnT (0) TnI (0); WCC (+).
Sonnino *et al.*^81^	R, DB, PC. *ex vivo*	17	AMI	Anakinra	100 mg	3 months	IL6 (+).
Abbate *et al.*^82^ (VCU-ART)	R, DB, PC	10	AMI	Anakinra	100 mg sc daily for 2 weeks	3.5 months	CI (+); LVEDV (+); LVEF (0); LVESVi (+); CRP (+).
Ridker *et al.*^83^	R, DB, PC	556	T2DM with high-MI risk	Canakinumab	5 OR 15 OR 50 OR 150 mg	4 months	CRP (+); fibrinogen (+); HbA1c (0); IL-6 (+); triglyceride (−).
**IL-6 inhibitors**							
Holte *et al.*^84^	R, DB, PC	117	NSTEMI	Tocilizumab	280 mg IV	6 months	Coronary flow reserve (0); VCAM-1 (+)
Kleveland *et al.*^85^	R, DB, PC	117	NSTEMI	Tocilizumab	280 mg IV	6 months	LVEF (0); SAE (0); nt-proBNP (0); Hb (0); hs-CRP (+); leukocytes (+); lipids (0); platelets (0); hs-TnT (+).
Carroll *et al*.^86^	R, DB, PC	28	MI	Tocilizumab	162 mg sc	1 month	MACE (0); CRP (0); ECG (0).
**TNF inhibitors**							
Padfield *et al.*^87^	R, DB, PC	26	MI	Etanercept	10 mg IV	24 h	IL-6 (+); neutrophil (+); platelet-monocyte aggregate (−); t-PA (0).
Mann *et al.*^88^ (RENAISSANCE)	R, DB, PC	1123	CHF (16.3%)	Etanercept	25 mg sc BIW OR TIW	24 weeks	Mortality (0); MACE (0).
Mann *et al.*^89^ (RECOVER)	R, DB, PC	925	CHF (20%)	Etanercept	25 mg sc QW OR BIW	5.5 months	Mortality (0); MACE (0);
Chung *et al.*^90^ (ATTACH)	R, DB, PC	150	CHF	Infliximab	5 or 10 mg/kg	28 weeks	Clinical composite score (0), LVEF (0), CRP (+), IL-6 (+), worsened heart failure (+), mortality (+)
Bozkurt *et al.*^91^	R, DB, PC	47	HF (82%)	Etanercept	5 mg/m2 OR 12 mg/m2 sc BIW for 3 months	3 months	Adverse events; functional status (0); LVEF (+); LVESV (+); LVEDV (+); LVESV (+) LV mass (0).
Fichtlscherer *et al.*^92^	ND, OL, NPC	18	HF (50%)	Etanercept	25 mg sc QW	7 days	Endothelium dependent forearm blood flow (+); endothelium independent flow (0)
Deswal *et al.*^93^	R, DB, PC	18	HF (83%)	Etanercept	1, 4, 10 mg/m^2^	2 weeks	EF (+); 6MWT (+), QOL scores (+).

ACS, acute coronary syndrome; T2DM, Type 2 diabetes mellitus; NSTEMI, non-ST elevation myocardial infarction; sc, subcutaneous injection; BIW, twice weekly; TIW, three times a week; QW, once weekly; IpPLA2, lipoprotein-associated phospholipase A2; MPO, myeloperoxidase; Ntpro-BNP, N-terminal pro b-type natriuretic peptide; TnI/T, troponin I/T; FBC, full blood count; WCC, white cell count; LVESVi, left ventricular end-systolic volume index; HbA1c, haemoglobin A1c; VCAM-1, vascular cell adhesion protein 1; Hb, haemoglobin; SAE, serious adverse events; t-PA, tissue plasminogen activator; EF, ejection fraction; R, randomized; DB, double blind; PC, placebo controlled; MACE, major adverse cardiac event; hs-CRP, high-sensitivity C-reactive protein; LVEF, left ventricular ejection fraction; TNF, tumour necrosis factor; ECG, electrocardiogram; (+), supports intervention use; (−), supports placebo use; (0), no difference between intervention and placebo; 6MWT, 6 min walk test; LVEDV, left ventricular end-diastolic volume; LVESV, left ventricular end-systolic volume.

##### Interleukin-6

IL-6 may have both pro- and anti-inflammatory properties and can activate the PI3K pathway, which is associated with adaptive hypertrophy and protects cardiomyocytes from apoptosis.^94^^,^^95^ IL-6 levels post-MI correlate with increased CRP production, which is a predictor of progression and future risk of coronary heart disease.^96^

Tocilizumab is an IL-6 receptor antagonist used as anti-inflammatory therapy for rheumatoid arthritis.^97^ A recent study by Kleveland *et al.*^85^ in 117 non-ST elevation MI (NSTEMI) patients undergoing coronary angiography showed significantly lower levels of high-sensitivity CRP (hs-CRP) and cTnT after tocilizumab administration over 3 days hospitalization. This was particularly substantial in acutely treated patients and during percutaneous PCI, which suggests cardio-protective potential during I/RI. A subsequent study by Holte *et al.*^84^ found no difference in coronary flow reserve (CFR), as a reflection of endothelial function, in these patients over 6 months follow-up, suggesting tocilizumab influences systemic inflammation as measured by CRP, not coronary endothelial function. The recent STAT-MI trial ended early as futility analysis showed low likelihood of reaching the primary outcome, with authors attributing early termination to insufficient subject recruitment. The results for 28 patients showed no significant change in MACE, CRP, or ECG.^86^^,^^98^

##### Interleukin-1

IL-1α is a cytokine released from necrotic cardiomyocytes and acts as a danger signal triggering acute inflammation and healing following MI.^99^ IL-1β is released by activated leucocytes within hours post-MI and has been implicated in leucocyte infiltration and ventricular remodelling.^100^^,^^101^ Importantly, pre-clinical data showed that antibody-mediated IL-1β neutralization reduced post-MI inflammation, adverse remodelling, and HF in mice.^102^^,^^103^

Anakinra is a recombinant competitive non-specific IL-1 receptor antagonist (IL-1RA) and has been used for the treatment of rheumatoid arthritis.^104^ Clinical trials using anakinra have shown significant down-regulation of systemic inflammation as measured by CRP in acute decompensated HF,^79^ NSTEMI ACS^80^ and AMI.^82^ Sonnino *et al.*^81^ also reported decreased IL-6 levels 3 months after anakinra treatment in AMI. In a small pilot study, Abbate *et al.*^82^ randomized 10 patients to either anakinra or placebo. Over a 10–14 week follow up period, improvements in LV remodelling with a drop in LV end-systolic volume index (LVESVi) were measured by cMRI. However, large and adequately powered studies are required to assess the safety and efficacy of anakinra, as Van Tassell *et al.*^79^ did not show a reduction in MACE up to two weeks after treatment with anakinra in 30 HF patients and another study (Morton *et al.*^80^) showed increased markers of inflammation and re-infarct rates following discontinuation of anakinra.

Canakinumab is a monoclonal antibody that specifically neutralizes the pro-inflammatory effects of IL-1β.^105^ Ridker *et al.*^83^ tested canakinumab treatment in 556 type-2 diabetes patients at high risk of MI and found an improvement in overall inflammatory measures. Recently the CANTOS trial (2017) studied the effect of canakinumab in 10 061 patients with previous MI and raised CRP levels. Canakinumab significantly reduced CRP levels compared with placebo, and it also significantly lowered the incidence of recurrent non-fatal MI, non-fatal stroke, and cardiovascular-related death after 2 years follow-up.^78^

**Figure 3:**
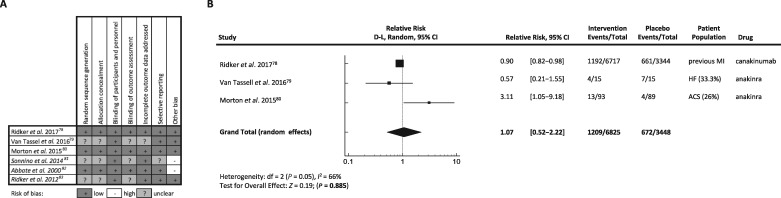
Meta-analysis of clinical trial outcomes of IL-1 inhibitors. (*A*) Risk of bias summary: review authors’ judgement about each risk of bias item for each included study. (*B*) Forest plot showing proportions of mortality rates, RR and 95% CIs for trials of IL-1 inhibition in MI and HF. The random effects model was used, and RR was determined using the DerSimonian–Laird method.

The above studies were analysed for their risk of bias profiles (*Figure 3A*). In two studies, we identified ‘other bias’, which is defined as ‘sources of bias relevant only in certain circumstances or not covered by the other headings’. Sonnino *et al.* (2014) was classified as such because studies were performed on *ex vivo* peripheral blood leucocytes.^81^ Abbate *et al.* (2010) was excluded, because it was a small pilot study with only 10 patients with a greater risk of strong impact by outliers.^82^ Both studies, as well as Ridker *et al.* (2012) did not measure MACE, so were excluded from meta-analysis.^83^ The three remaining trials measured MACE and were included in meta-analysis.^78–80^ Of 10 273 patients, 30 were classified as HF and 10 243 as high risk of HF. The risk ratio (RR) and confidence intervals (CIs) for mortality and MACE were calculated as 1.07 (0.52–2.22) showing that overall mortality and MACE in the treated groups was not decreased compared to the placebo groups (*Figure 3B*). However, these studies still present a significant heterogeneity in study design and population (secondary prevention in patients at high risk of MI, HF, or ACS patients) and tested drug (canakinumab or anakinra).

The CANTOS study was still a major step forward investigating a more targeted approach to immunomodulation following MI. It was however, primarily focused on preventing atherosclerosis and results are assumed to be due to a reduction of systemic inflammation, and subsequently less thrombosis-mediated cardiovascular events. Measurements of cardiac functional or morphological parameters would have allowed a conclusion about direct effects on the heart.

##### TNF

TNF promotes leucocyte infiltration by up-regulating transcription of adhesion molecules and chemokines. However, TNF also demonstrates pleiotropic effects in delaying myocyte apoptosis following acute ischaemia.^106^ Etanercept is a high affinity TNF receptor which has been licensed for treatment of rheumatoid arthritis.^107^ A small early study of etanercept by Deswal *et al.*^93^ given to 12 HF patients appeared to improve ejection fraction and 6MWT performance over placebo control subjects. In another study by Fichtlscherer *et al.*,^92^ etanercept treatment of 13 CHF patients seemed to increase endothelium-dependent forearm blood flow as compared with 5 control patients, but not endothelium-independent, suggesting improvement in systemic endothelial vasoreactivity. Bozkurt *et al.*^91^ performed a trial using biweekly subcutaneous injections of etanercept 5 mg/m^2^ (*n* = 16) or 12 mg/m^2^ (*n* = 15) or with placebo (*n* = 16) for 3 months in patients with NYHA Class III to IV HF and showed dose-dependent improvement in LV structure and function, with improved NYHA class when compared to placebo.

During the RECOVER and RENNAISANCE studies, NYHA Class III to IV CHF patients received placebo (*n* = 373) or 25 mg of etanercept once, twice, or three times per week (*n* = 375 each group). No clinical or survival improvements were observed after nearly 2 years, and therefore, both studies were prematurely terminated.^88^ Joint analysis (RENEWAL) of both trials rules out a clinically relevant benefit of etanercept on the rate of death or hospitalization due to CHF.^89^

A final trial using etanercept by Padfield *et al.*,^87^ in a 26 patient AMI study also failed to show beneficial clinical effects and found that in fact reductions in neutrophil and IL-6 levels correlated with increased platelet and monocyte activation.

Another TNF inhibitor, the chimeric monoclonal antibody infliximab, was used at 5 or 10 mg/kg at 0, 2, and 6 weeks in a trial on 150 patients with moderate-to-severe HF, but no improvement in clinical status was observed despite suppression of inflammatory markers CRP and IL-6. However, there was a higher ratio in adverse effects noted in the 10 mg/kg infliximab group.^90^

The original rationale behind targeting inflammatory cytokines as preventive strategy as well as post-MI stems largely from the emerging concept of atherosclerosis as an inflammatory disease. Preventing an exacerbation of atherosclerosis by targeted anti-inflammatory treatments, previously shown to be effective in systemic autoimmune disease, was anticipated to protect from recurrent cardiovascular events. Importantly however, besides decreasing systemic inflammation, studies on IL-1 receptor antagonist anakinra suggest the potential for effects on heart tissue function and remodelling.^82^ This allows a first promising glimpse into the potential benefit of these therapies in protecting from adverse remodelling and development of HF post-MI.

## 4. Considerations for future research and trial design

An enormous amount of effort and resources has gone into a wide range of immunomodulatory trials (*Figure 1*), which have accumulated a wealth of invaluable information to allow future trials to be designed in the most efficient way. Yet to date, practical challenges dealing with a highly diverse post-MI patient population, and striking heterogeneity in trial design means evidence available in support of immunomodulatory treatment is still inconclusive. We recommend consideration of the following factors to achieve standardization in the design of future clinical trials.

### 4.1 Therapeutic strategy and target

Due to the dynamic nature of the post-MI immune response, small variations in exact target, timing, and dosage can lead to tremendous differences in the effect on the immune response. In addition, both pre-MI and early post-MI clinical situation affect long-term complications, including the development towards HF. Early and late phase post-MI should therefore not be looked at in isolation. When interpreting the currently available information on immunomodulation trials, it is crucial to discriminate between studies on pre-MI prevention, AMI recovery, or CHF mortality. Most trials so far have focused on AMI, thus investigated early effects only. Future trials need to carefully consider the best time point for drug administration to optimize acute infarct healing and long-term functional recovery.

### 4.2 Routine medication/therapy

Currently used routine post-infarct medication affect the immune response, and this may have significant impact on experimental treatments and may result in unexpected outcomes if routine treatment is changed. There are several prominent examples of routine medication with either pro- or anti-inflammatory effects. Platelet inhibitors such as aspirin and clopidogrel attenuate platelet-leucocyte interaction, thus suppress ROS formation, leucocyte activation, and expression of pro-inflammatory cytokines.^108^^,^^109^ Statins, which are still used to block the production of low-density lipoprotein (LDL) cholesterol, down-regulate effector T lymphocytes, while increasing expression of Treg cell markers, IL-10, and tumour growth factor-β (TGF-β).^110^ On the other hand anti-hypertensive beta-blockers such as metroprolol and propranolol reduce IL-10 and TGF-β levels^111^ and information on the immunological effects of vasodilators such as angiotensin conversing enzyme inhibitor (ACEi) has been conflicting.^112^ Accordingly, depending on standard treatment regime, immunomodulatory therapy may be hampered or boosted.

### 4.3 Patient population

Trial results also hint towards the need of a stratified approach to immunomodulatory care of post-MI patients, as some patient groups may respond and benefit significantly more than others. The post-MI population is strikingly heterogeneous and the risk of poor outcome and development to HF is influenced by several factors including demographics, genetics and pre-existing immunological, and metabolic conditions.^113^ It seems feasible that immunomodulatory treatment may be most beneficial for patients with a raised inflammatory profile and the CANTOS trial has indeed provided first evidence for this hypothesis.^78^

### 4.4 Combination therapy

While the immune response is certainly a crucial player in post-MI wound closure and restoration of tissue integrity, its modulation will likely only prevent ongoing damage rather than replacing tissue already lost. Long-term benefits on heart function are more likely to be achieved if immunomodulation is combined with regenerative therapies such as biomaterial-based strategies or cell therapies aiming to replace damaged cardiomyocytes. Combination therapy will not only prevent ongoing damage, but also provide therapies with a local environment permissive of regeneration.^6^ Numerous studies have been undertaken in recent years to evaluate the effect of stem cell mobilization, recruitment or transplantation into the myocardium.^114^^,^^115^ One approach particularly interesting in this context is the use of mesenchymal stem/stromal cells (MSC). MSC themselves combine cell therapy with immunomodulation due to their stem cell-like and immunosuppressive characteristics. Although it has become clear that their initially assumed immunoprivilege may be lost upon differentiation, a Phase I/II randomized comparison (POSEIDON study) of trans-endocardial injection of MSCs in patients with LV dysfunction showed positive effects on functional capacity, quality of life, and ventricular remodelling.^116^ Once both immunomodulatory strategies and cardiac regenerative cell therapy have overcome current hurdles, the combination might be able to achieve astonishing results.

### 4.5 Complications of immunotherapies

Cost-benefit ratios and incidence of severe side effects need to be carefully considered and investigated before administration of post-MI immunotherapies. A tightly regulated balance between immune activation and regulation ensures efficient responses against infections and cancers, while preventing autoimmunity. Suppressing all or part of the immune system post-MI may impact on wound healing and regenerative processes in other areas of the body, weaken defences against infectious diseases, and undermine the ability of the immune system to detect and destroy malignant growth early on. Chronic immunosuppressive treatment using methotrexate, cyclosporine, or anti-TNF reagents is known to carry an increased risk of lymphoma, due to decreased immunesurveillance.^117^ Importantly however, these potentially serious complications need to be assessed for individual treatments and will depend on the specific role of the immunomodulation target in the relevant settings. For example, IL-1β inhibition with canakinumab during the CANTOS trial reduced the incidence of lung cancer and lung cancer mortality, while fatal infections and sepsis were more common in the canakinumab treated group.^118^

## 5. Recommendations for standardization of future post-MI immunomodulatory trials

### 5.1 Start of treatment

To ensure the initiation of an appropriate wound healing response, administration of strong immunosuppressive treatment within 24 h after AMI should be avoided. Two to 3 days post-MI may be a more beneficial time point causing less adverse effects. This may be hard to achieve with strategies targeting events or factors immediately post-MI (e.g. ROS, complement). Notably, due to its effects on the adaptive immune system, CsA which has been used safely (albeit without clinical effects) at early time points, with the aim to prevent immediate post-MI cardiomyocyte death, may be a worthwhile candidate to explore for its long-term effects on HF development. Trials targeting mast cells have also yielded promising results and they may benefit from a wider therapeutic window, allowing treatment to be delayed for a few days post-MI.

So far, the maybe greatest patient benefit of immunomodulatory treatment was achieved by IL-1 inhibition (CANTOS trial) in previous MI patients with raised inflammatory profiles, thus at greater risk of developing subsequent MIs or HF. Due to the delay post-MI, this patient population was likely beyond the acute phase of wound healing and inhibition of a potent inflammatory mediator successfully decreased systemic inflammation without affecting early healing.

### 5.2 Outcome measures

Besides MACE, outcome measures to allow an assessment of both acute and long-term benefits of immunomodulatory treatment should include functional and morphological measurements such as LV ejection fraction and diameters as assessed by echocardiography or MRI. Importantly, these should be obtained at baseline and at defined time points following AMI (e.g. 1 month, 3 months, 1 year, 2 year, and 4 year following MI). Considering that 63% of MI patients develop HF within 6 years post-MI,^2^ shorter follow up periods will not allow to judge if the treatment affects development towards HF. Incorporating measurement of CRP and BNP or NT-pro BNP will help identify patients’ response to therapies and identify patients at risk of overt immune suppression. Measuring adaptive immune auto-reactivity against the heart (e.g. serum auto-antibody levels) would further improve our understanding of the immunological factors involved in post-MI HF development.
